# Role of the Osmotic Stress Regulatory Pathway in Morphogenesis and Secondary Metabolism in Filamentous Fungi

**DOI:** 10.3390/toxins2040367

**Published:** 2010-03-24

**Authors:** Rocio Duran, Jeffrey W. Cary, Ana M. Calvo

**Affiliations:** 1 Department of Biological Sciences, Lincoln Hwy. 1425W., Montgomery Hall, Northern Illinois University, DeKalb, IL 60115, USA; 2 Food and Feed Safety Research Unit USDA, ARS, Southern Regional Research Center, 1100 Robert E. Lee Blvd, New Orleans, LA 70124, USA; Email: jeff.cary@ars.usda.gov

**Keywords:** *Aspergillus*, osmotic stress, regulation, morphogenesis, secondary metabolism

## Abstract

Environmental stimuli trigger an adaptative cellular response to optimize the probability of survival and proliferation. In eukaryotic organisms from mammals to fungi osmotic stress, mainly through the action of the high osmolarity glycerol (HOG) pathway, leads to a response necessary for adapting and surviving hyperosmotic environments. In this review we show that the osmoadaptative response is conserved but not identical in different fungi. The osmoadaptative response system is also intimately linked to morphogenesis in filamentous fungi, including mycotoxin producers. Previous studies indicate that the response to osmotic stress is also coupled to the biosynthesis of natural products, including mycotoxins.

## 1. Introduction

Filamentous fungi are ubiquitous in nature, capable of inhabiting very diverse ecological niches. Many filamentous fungi propagate by means of dispersal of asexual spores termed conidia. Spores can traverse very long distances through the air which allows them to come to rest in environments that can be very different from that of their origin. In order for the fungus to proliferate and thrive in nature, it must be capable of adapting quickly to any particular environment it may find itself in. Even when acclimated to their particular niche, perturbations can occur from time to time in the surrounding environment either as a result of natural causes (*i.e.*, drought) or man-made causes (*i.e.*, modern agriculture). To adapt to such environmental changes fungi must continually adjust their physiology in order to enhance survival. At the molecular level, fungi possess complex signal transduction pathways that allow the fungus to respond appropriately to alterations in external stimuli. These stimuli can include biotic stress, and abiotic stresses such as changes in temperature, pH, nutrient availability, oxidative stress and osmotic stress. In this report we will be focusing on the role that osmotic stress plays in the growth and development of filamentous fungi and the molecular mechanisms that control the fungus’ response to alterations in osmotic pressure.

Many of the studies that first identified genes involved in the molecular regulation of cellular responses to osmotic pressure were performed in the yeast *Saccharomyces cerevisiae* (reviewed in [1]). These included the identification of MAP kinase signaling pathways and the high osmolarity glycerol (HOG) pathway involved in response to osmotic pressure [2]. In addition to providing information gleaned from studies using *S. cerevisiae*, we will also focus on research involving filamentous fungi, particularly of the genus Aspergillus. *A. nidulans* has been used extensively as a model fungus for the study of the molecular genetics of responses to osmotic stress due in large part to the availability of a vast number of genetic markers and a sexual stage that allows for genetic recombination studies [3,4]. *A. nidulans* also produces the mycotoxin sterigmatocystin, which is synthesized through the same conserved pathway that leads to production of aflatoxin in other Aspergilli. Both compounds are potent carcinogenic mycotoxins. In addition to A. nid*ulans*, the genus *Aspergillus* includes a number of species that are of great importance both economically and medically. For example, the agriculturally important aflatoxin-producing species *A. flavus* and *A. parasiticus* [5]; *A. oryzae* and *A. niger* as industrially important sources of enzymes [6,7]; and medically important species such as the causal agent of human aspergillosis, the gliotoxin-producer *A. fumigatus* [8]. A number of studies have reported on the effects of osmotic stress on *Aspergillus* spp. as influenced by water content, water activity (Aw), and solute concentrations. Lillehoj *et al.* [9] reported on the effect of moisture (measured as percent moisture content or osmotic pressure) and substrate variation in developing cottonseed and corn. They found that osmotic pressure and other factors such as oil and starch content of seeds determine the physiological responses of *A. parasiticus*. Maximum accumulation of aflatoxin was observed in corn kernels and cottonseed that were inoculated at 52 and 70% moisture content, respectively. A water availability in the 600 kPa range of osmotic pressure provided optimum conditions for *A. parasiticus* development in corn kernels. Osmotic stress has also been shown to be a critical factor in enzyme production, relevant in industrial fermentations that utilize aspergilli. It was found that increased osmotic pressure as determined by NaCl concentration resulted in increased production and secretion of glucose oxidase during fermentation by *A. niger* [10]. Kobayashi *et al.* [11] found that glucoamylase activity by *A. oryzae* showed an increase of about 20-fold as water content of the wheat bran substrate was increased.

Scientists have made great strides in elucidating some of the basic molecular mechanisms that allow fungi to survive and proliferate under different environmental conditions. The fact that osmoadaptation is easy to manage and mimic in a lab setting has aided in studies of fungal responses to osmotic stress both at the cellular and molecular level. The advent of whole genome sequencing and functional genomics has also provided another tool to dissect the regulatory factors and signal transduction pathways that respond to osmotic stress. A recent review of annotated stress proteins by Miskei *et al*. [12] identified a number of genes in different *Aspergillus* species (*A. nidulans, A. flavus, A fumigatus, A. niger, A. terreus, A. oryzae, A clavatus* and *N. fisheri)*, that are orthologous to those encoding components of the *S. cerevisiae* HOG pathway [2]. These studies have facilitated the generation of *A. nidulans* strains that harbor mutations in several members of the HOG pathway [13,14]. Miskei *et al.* [12] also proposed in this report that the response of the HOG pathway in filamentous fungi is not exclusively due to the fungus’ response to osmotic stress but also to oxidative stress. In this review we strive to present a compilation of information on the genes involved in the molecular regulation of the response to osmotic pressure in filamentous fungi mainly from studies with the model fungus *A. nidulans* and the model yeast *S. cerevisiae*. We also include an overview of the effect of osmotic stress on development and secondary metabolism in filamentous fungi, particularly those associated with mycotoxin production. In addition, we present information from our labs on the velvet gene (*veA*) and its role in filamentous fungi as a light-responsive factor that can integrate external stimuli, including osmotic stress to bring about physiological responses that are often manifested as alterations in secondary metabolism and/or morphogenesis. 

## 2. Osmoadaptation Mechanisms in Yeast

**Figure 1:**
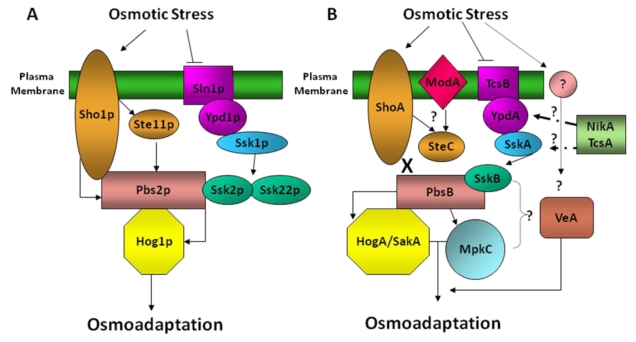
Comparison of *S. cerevisiae* (A) and *A. nidulans* (B) HOG regulatory pathways.

Numerous studies conducted in the yeast *S. cerevisiae* have contributed to the understanding of the high osmolarity glycerol (HOG) pathway (reviewed in [1]). Activation of the HOG pathway depends on an increase in osmotic pressure resulting in a change in the expression of several genes. The molecular response to osmotic stress in *S. cerevisiae* is mediated by mitogen-activated protein kinase (MAPK) signaling pathways. Hog1p MAPK plays a central role in this signaling system, where Hog1p is activated by a two-component His-to-Asp phosphorelay system that includes Sln1p, a histidine kinase sensor; Ypd1p, a histidine-containing phosphotransfer protein; and Ssk1p and Skn7p response regulators [15,16] (Figure 1). Sln1p protein consists of an extracellular sensor, a kinase and a response regulator domain, giving a transmembrane hybrid-type histidine kinase. Under low-osmolarity, a specific histidine residue (His576) within the histidine kinase domain is autophosphorylated. Then, the phosphate group of the histidine kinase domain is transferred to an aspartate residue (Asp1144) within the receiver domain of Sln1p. Through a His-Asp phosphorelay, the phosphate group is transferred to the downstream Ypd1p phosphotransmitter, and then to the Ssk1p response regulator [12,15,17]. Phosphorylated Ssk1p fail to interact with the redundant pair of MAPKKK Ssk2p/Ssk22p, resulting in an inactive Ssk2p/Ssk22p-Pbs2p (MAPKK)-Hog1p form [18]. However, when yeast are exposed to osmotic stress, Sln1p is inhibited resulting in dephosphorylated forms of Ypd1p and Ssk1p, leading to Ssk1p-Ssk2p/Ssk22p interactions and finally the activation of Hog1p by phosphorylation [18]. In hyperosmotic environments, activated Hog1p migrates to the nucleus. This action is mediated by Gsp1p, a small GTP-binding protein, and the importin homologue Nmd5p; and it is independent of the NLS-binding importin/heterodimer [19]. Activation of the HOG pathway leads to the induction of genes required for osmotic stress response, for example glycerol biosynthesis genes such as the genes encoding a glycerol-3-phosphate dehydrogenase (GPD1) and glycerol-3-phosphatase (GPP2) [20,21].

A nuclear exchange sequence receptor protein called Crm1p exports dephosphorylated Hog1p back to the cytoplasm [19]. Negative regulation of Hog1p is exerted through phosphotyrosine phosphatases, Ptp2p and Ptp3p, in both the nucleus and the cytoplasm, respectively [22]; and the phosphatase Ptc1p, Ptc2p and Ptc3p [23,24]. Ptc1p dephosphorylates Hog1p *via* a docking interaction between Ptc1p, Hog1p, and Pbs2p joining a small adaptor named Nbp2p [25,26].

In addition, in yeast Hog1p is regulated by a second mechanism, which includes a transmembrane sensor kinase called Sho1p (Figure 1). In the Sho1 branch, a multi-component signaling complex consisting of Sho1p, Cdc42p, Ste20p/Cla4p, Ste50p, Ste11p (MAPKKK), Pbs2p is formed in hyperosmotic environments. Pbs2p is localized to the membrane by its proline-based motif and an SH3 domain on Sho1. Pbs2p binds the MAPKKK Ste11p which is phosphorylated by the PAK-like kinase Ste20p, which is itself recruited to the membrane by activated Cdc42. Activated Ste11p then phosphorylates Pbs2p which in turn activates the downstream Hog pathway [26,27]. Hkr1p and Msb2p, mucin-like transmembrane proteins [27], are potential sensors for the Sho1p mechanism that activates Hog1p when the yeast is exposed to osmotic stress.

Activation of Hog1p can also lead to phosphorylation of the Sko1p bZip-type repressor, which results in the disassembly of Sko1p-Tup1p-Ssn6p repressor complexes [28]. Phosphorylated Hog1p interacts with the RCS chromatin-remodeling complex to mediate its recruitment to osmo-responsive genes [29]. Hog1p also recruits the Rpd3p histone deacetylase to osmoresponsive gene promoters inducing their expression during osmotic stress [30]. Hog1p is necessary for the activation and increase of the RNA polymerase II complex and mRNA, behaving as a transcriptional elongation factor specific for genes induced upon osmotic stress [29,31]. Phosphorylated Hog1p is also necessary for the activation of Msn2p/Msn4p C2H2 zinc finger transcription factors involved in general stress response [32,33,34,35].

Other Hog1p targets include Sgd1p, a novel essential nuclear protein. *SGD1* was identified as a high-copy-number suppressor of the osmosensitive phenotype of pbs2D and hog1D deletion mutants [36,37]. In addition, Hog1p interacts and phosphorylates the transcription factor Smp1 upon osmotic stress controlling a subset of the responses induced by the MAPK [38]. Activated Hog1p can phosphorylate the Rck2p protein kinase, which participates in G2 checkpoint control and the osmotic stress triggered attenuation of protein synthesis [39,40]. 

In addition to the HOG signaling pathway, c-AMP dependent kinases have been shown to affect gene expression under osmotic stress [41]. However, this response is not exclusive to osmotic stress but a general stress response to a variety of additional stimuli such as nutrient starvation, heat shock, and oxidative stress among others [42,43,44]. Osmotic shock can also stimulate the production of compounds such as phosphatidylinositol-3,5-bisphosphate, which could participate as a second messenger in the activation of the osmotic signaling response [45].

## 3. Hog Pathway in *Aspergillus nidulans*

As in the case of *S. cerevisiae*, the HogA (SakA) pathway of *A. nidulans* (Figure 1) is activated in the osmotic [13,14,46,47,48] and oxidative [46,47] response by the *A. nidulans* Ssk1p ortholog, SskA. Interestingly, a *sakA* null mutant presented only slight sensitivity to high osmolarity stress, indicating that osmoregulation in *A. nidulans* differs from that described in yeast [14,46]. Other upstream components of this pathway are the Sln1p homolog, TcsB [49]; the Ypd1p ortholog, YpdA [14,48]; and also NikA, a dispensible Mak2-type histidine kinase in the osmotic stress response that has been demonstrated to transmit fungicide-induced stress signals such as those generated in the presence of fludioxonil and iprodione [48,50] (Figure 1). A *tcsB* deletion (*tcsB*) mutant did not present an osmosensitive phenotype [51] most likely due to redundancy of function with other histidine kinase genes present in *A. nidulans* genome [48,52]. However, the *A. nidulans* TcsB–YpdA–SskA system might have a role similar to that of Sln1p–Ypd1p–Ssk1p proteins in yeast [14]. A non-essential sensor kinase, TcsA, may be involved in conidiation [53] however, a TcsA–YpdA–response regulator signalling pathway has not been demonstrated. 

Interestingly, it is likely that the Sho1p signaling pathway in *A. nidulans* might not be involved in osmoregulation but instead may be carrying out other signaling functions [54]. *A. nidulans* PbsB (homolog to *S. cerevisiae* Pbs1p) lacks a typical Pro-rich motif necessary for the binding with Sho1p [14,55]. Furthermore, proteins such as ModA, a Cdc42p-like protein [56], similar to Sho1p, have a role in morphogenesis. SteC, similar to Ste11p, regulates sexual development [57]. Differently from yeast, *A. nidulans* PbsB MAPKK activates another Hog1p ortholog, MpkC, not present in yeast. Although MpkC is not necessary for osmoregulation, overexpression of *mpkC* suppresses the slight high-osmolarity sensitivity of *hogA* strains [14]. An *A. nidulans* orthologue of *S. cerevisiae* Skn7p, SrrA, plays a role in both oxidative stress and osmotic stress resistance [47,48].

Little is known about SakA and YpdA import/export to the nucleus, although putative orthologues of Gsp1p (RanA), Nmd5p importin and Crm1p (KapK) export factor have be identified [12]. Dephosphorylation of HogA/SakA in *A. nidulans* is mainly unknown, except for the up-regulation of *ptpA* (encoding a putative protein phosphatase) when the fungus is exposed to osmotic stress [13]. Several SakA-dependent regulators have been identified, such as RpdA (Rpd3p ortholog), AtfA (putative ortholog of *S. pombe* Atf1) [58,59], and RcoA (*S. cerevisiae* Tup1 ortholog). Msn2p/Msn4p-type protein, MsnA, has been found in *A. nidulans*, and was shown to be induced by different types of stress [13]. In *A. nidulans*, potential target genes of SakA and SrrA (*S. cerevisiae* Skn7p homolog) includes *gfdB* (*S. cerevisiae* GPD2 ortholog) [13,60] and *enaA* [13]. 

The process of osmotic adaptation by activation of the HOG pathway, in both yeast and filamentous fungi, results in the biosynthesis and accumulation of compatible molecules such as proline, trehalose, polyols and glycerol to counterbalance the osmotic pressure and prevent loss of water. In *A. nidulans*, SskA (*S. cerevisiae* Ssk1p homolog) regulates the expression of genes involved in conidial tolerance to stress, including genes involved in glycerol and trehalose metabolism (*gfdA* and *gfdB*, glycerol-3-phosphate dehydrogenases; *gldB*, glycerol dehydrogenase; *tpsA*, trehalose-6-phosphate synthase; *orlA*, trehalose-6-phosphate phosphatase; *treB*, neutral trehalase) [13,61,62,63,64,65].

## 4. Examples of the Osmoadaptation Signaling Pathway in Other Aspergilli

Regulatory pathways including those signaling pathways responsive to stress are relatively conserved, but not identical, in the genus Aspergillus [12]. For example, conserved SakA plays an important role in the oxidative stress response in the opportunistic human pathogen and gliotoxin-producer *Aspergillus fumigatus*, while the TcsB histidine kinase was not crucial [66]. In *A. fumigatus*, MpkC plays other roles such as in carbon source utilization, however it was not determined to be involved in osmoadaptation [67]. *Aspergillus niger*, an important citric acid, gluconic acid and hydrolytic enzyme producing fungus, does not have TcsB-type histidine kinase homologs. Other aspergilli, such as *A. nidulans* mentioned above, present TscB but it is dispensable in the osmoadaptation response. It is likely that TcsB is dispensable or not present in other aspergilli [51,66]. 

Interestingly, the important aflatoxin-producer *Aspergillus flavus* harbors two orthologs of the *A. nidulans* SskA response regulator in its genome (ORFs AFL2G_06337 and AFL2G_12585), and two orthologs of the *A. nidulans* ortholog RpdA histone deacetylase (ORFs AFL2G_08263 and AFL2G_03062). This suggests that *A. flavus* has a more sophisticated osmotadaptation system compared to other aspergilli that may include genes with redundant functions.

## 5. Role of the Osmotic Stress-response Pathway on Fungal Development and Secondary Metabolism

The study of the HOG pathway and its implications in morphological development and secondary metabolism in filamentous fungi are still at the earliest stages. In *A. nidulans*, a *sakA* deletion mutant shows development and cell-specific phenotypes [46]. This mutant is characterized by premature sexual development. In addition, deletion of *sakA* results in conidia that are highly sensitive to oxidative and heat shock stress and lose viability overtime. This evidence supports the role of SakA as a repressor of sexual development and asexual spore stress resistance and survival. As mentioned in section 3, another member of the *A. nidulans* HOG pathway, SskA is important in regulating the tolerance of conidia to osmotic stress. SakA was also shown to be involved in conidia viability in *A. fumigatus*, where the *sakA* mutant presented alterations in conidia germination in a nitrogen source-dependent manner [68]. The bZip-type transcription factor AtfA (putative ortholog of *S. pombe* Atf1), that functions downstream of the HogA MAPK in the course of fludioxonil and osmotic stress response, is also involved in conidia stress tolerance [58,59,69,70]. Also in *A. fumigatus*, mutations in MA21, a homolog of the *S. cerevisiae* transmembrane sensor kinase *SHO* gene, showed a reduction in growth and germination rates. It demonstrated irregular hyphal morphology with a decrease in phialides and conidia [71]. Development is also affected by osmoregulation in dimorphic fungi such as *Candida albicans*, where the HOG homolog is necessary for chlamydospore formation [72]. 

Additionally, studies on *A. nidulans* SteC revealed its role in morphogenesis. Deletion of the *steC* gene results in a slower growth rate, increase of branched hyphae, alteration of conidiophore morphology, inhibition of heterokaryon formation and a blockage of cleistothecial development. The gene is transcriptionally activated during asexual development and controls the phosphorylation of two putative MAP kinases [57]. Also, RcoA (homolog of *S. cerevisiae* Tup1p) affects growth and sexual/asexual development in *A. nidulans* [73,74]. The response regulators SrrA and SskA are not only involved in osmotic and oxidative stress signal transduction but also in regulation of asexual development in *A. nidulans*. Deletion of any one of these genes results in a reduction in conidiation and spore viability [48].

The evidence provided by *A. nidulans* studies as well as studies in other fungi clearly indicates a connection between the osmotic stress regulatory system and fungal development. Recently in our laboratories we found that osmotic stress caused by high concentrations of sodium chloride, sorbitol or potassium chloride positively affects vegetative growth leads to an increase in conidiation in *A. flavus* (Duran *et al.*, manuscript in preparation). Han *et al*. [75] also reported an increase of conidiation in the presence of high amount of sorbitol in *A. nidulans*. In contrast, in a study by Mert and Ekmekci [76] where *A. flavus* was inoculated on NaCl medium, a reduction of conidia was observed. The *A. flavus* strains are genetically diverse and they have been sub-divided into two strain types, S and L, based differences in sclerotial size [77]. It is possible that this variability in conidiation could be the result of different *A. flavus* strains used in these studies leading to different responses to osmotic stress and salinity. It is also possible that other culture conditions could have caused the observed differences in conidiation under induced osmotic stress. In addition, our recent study showed the light-dependent global regulator VeA, known to control sexual/asexual morphogenesis and secondary metabolism in filamentous fungi [78], to be associated with the osmotic-stress response in *A. flavus* (Duran *et al.*, manuscript in preparation and Figure 1). Specifically, osmostress enhances hyperconidiation in the *A. flavus veA* deletion strain, suggesting that *veA* is involved in modulating osmotic stress-induced conidiation. On the other hand, salinity stress has been shown to have an inhibitory effect on the formation of sclerotia in fungal species, including *Sclerotinia sclerotiorum*, *Rhizoctonia solani* and *Sclerotium rolfsii* [79]. In the mycotoxin producer *Aspergillus ochraceus* osmotic stress had little effect on sclerotia production, [80]. In *Botrytis cinerea* sclerotia production was also reduced as osmotic stress increased [81]. In *A. flavus*, hyperosmotic media caused a delay in sclerotial maturation (Duran *et al.*, manuscript in preparation). These studies suggest that under stress caused by hyperosmolarity the fungus favors investment of material and energy towards developmental programs (conidiation) that are conducive to survival *via* dissemination to a more favorable environment versus survival in the unfavorable hyperosmotic environment.

Previous studies support an association of fungal morphogenesis with secondary metabolism, including mycotoxin production [82]. Aflatoxin is one of the most potent natural carcinogenic compounds described. Surprisingly, hyperosmotic levels of NaCl, KCl or sorbitol did not affect the biosynthesis of this mycotoxin (Duran *et al.*, manuscript in preparation). Some osmotic stress genetic response elements have been demonstrated to be linked to toxin production in *A. nidulans*, specifically, RcoA (Tup1p homolog) affects not only fungal growth and development, but also is necessary for the production of sterigmatocystin in *A. nidulans* [83]. Additionally, osmotic stress does affect the production of other secondary metabolites in aspergilli, for example pigments. The *A. fumigatus sakA* deletion mutant is unable to produce pigmentation as observed in the wild-type strain, indicating that secondary metabolism is affected by the HOG signaling pathway in this fungus [68].

Across fungal genera, the role of MAPK cascades in the osmotic stress response is quite diverse. In the filamentous fungus *Fusarium graminearum*, a significant relationship between osmotic stress and secondary metabolism has been observed. *F. graminearum* is a common pathogen of grain producing crops. Among the several secondary metabolites generated by this fungus, the synthesis of trichothecene toxins and a reddish colored pigment called aurofusarin are affected by osmotic stress. Ochiai *et al.* [84] described the participation of several histidine kinases, components of the osmotic response signalling pathway, in the regulation of secondary metabolism of *F. graminearum.* These authors showed that production of trichothecenes is markedly suppressed by NaCl, without a significant effect on fungal growth. A null mutant of *FgOs1* (encoding the osmosensor histidine kinase) produced a reduced amount of the red pigment aurofusarin however it was unaltered in its ability to produce trichothecenes. Deletion null mutants of *FgOs4* (encoding MAPKKK), *FgOs5* (MAPKK), and *FgOs2* (MAPK) all showed markedly enhanced pigmentation and failed to produce trichothecenes, coinciding with a marked reduction of expression of *Tri4* and *Tri6* (trichothecene biosynthetic pathway and regulatory genes). In the maize pathogen, *Cochliobolus heterostrophus*, *hog1* mutants are more pigmented than the wild-type and demonstrate smaller appressoria and reduced virulence [85]. In *cpmk1* (*hog1* homolog) mutants of *Cryphonectria parasitica*, there was an increase in osmosensitivity along with reduced pigmentation, conidiation, and virulence on chestnut trees compared to a wild-type strain [86] In the rice pathogen, *Magnaporthe grisea*, *osm1* (*hog1* homolog) mutants were more sensitive to osmotic stress and showed some morphological defects. However, glycerol accumulation and appressorial turgor generation was unaltered compared to wild-type and virulence was not affected [87].

## 6. Conclusions

The ability to adapt is essential for filamentous fungi and yeast to survive and proliferate under non-optimal osmotic environments. Fungi sense and then transduce external changes in osmotic pressure mainly through cellular signaling pathways such as the HOG pathway that is mediated by MAPK cascades. Activated MAPKs phosphorylate a number of substrates including transcriptional activators that in turn modulate patterns of gene expression and subsequent protein synthesis. In fungi, responses to osmotic stress include the production of osmoprotectant compounds such as glycerol, reorganization of the cytoskeleton, and cell wall biogenesis. A fungus’ response to high osmotic pressures in an artificial environment such as during an industrial fermentation that utilizes controlled environmental and nutritional parameters is fairly straightforward. However, during a host-fungal pathogen interaction, the degree to which a fungus’ response to changes in osmotic pressure impacts its ability to successfully invade and survive in the host is not so clear. During the infection process, a pathogenic fungus would be expected to encounter a complex nutritional milieu upon lysis of plant cells that may include increased osmotic pressure. The entomopathogenic fungus, *Metarhizium anisopliae*, kills insects by direct penetration of the cuticle followed by multiplication in the hemolymph. The solute-rich hemolymph is characterized by high osmotic pressure [88] that could initiate a stress response in the fungus. *M. anisopliae* strains that were defective in the MOS1 osmosensor (SHO1 in yeast) displayed increased sensitivity to osmotic pressure as well as reduced virulence against larvae of *Manduca sexta* [89]. In the case of a plant necrotophic fungus, the breakdown of cell walls by fungal hydrolases and the generalized hypersensitive response by the host plant may result in the invading fungus being isolated in a region of high osmotic pressure. In addition, activation of the Hog1 MAPK pathway has been observed in *Candida albicans* cells exposed to the human antimicrobial peptide, histantin 5 [90]. It is possible that antimicrobial compounds elicited by the host may also trigger activation of the osmotic stress response in an invading plant or human pathogenic fungus leading to accumulation of osmolytes such as glycerol and reorganization of cell wall structure in an attempt to stave off death. With the ever increasing number of organisms whose genomes and proteomes have now been sequenced and annotated, it should be possible to better elucidate the impact that genes involved in the osmotic stress response have on host-fungal pathogen interactions. 

The literature cited in this review indicates that the osmoadaptative response is conserved but not identical in different fungi and that it is intimately linked to morphogenesis. The ability to alter morphogenic programs in response to changes in osmotic pressure can play a significant role in the fungus’ capacity to survive and flourish in nature. One developmental response to an increase in osmotic stress often observed in fungi is an increase in conidiation. This increases the fungus’ chances of survival as conidia can be dispersed by wind over wide areas thus removing the fungus from an inhospitable environment and potentially allowing it to take up residence in a more favorable environment. Furthermore, some of the studies mentioned in this review have also shown that in some cases the response to osmotic stress by fungi is linked to the biosynthesis of natural products, including mycotoxins. However, examples of this are few and more studies will be necessary to gain further insight into the interaction between osmotic stress regulation and secondary metabolism.
